# Diagnosis and treatment of a patient with Kimura’s disease associated with nephrotic syndrome and lymphadenopathy of the epitrochlear nodes

**DOI:** 10.1186/s12882-015-0007-7

**Published:** 2015-02-06

**Authors:** Sheng-lang Zhu, Peng-fei Wei, Jie-hui Chen, Zhen-fu Zhao, Qian-na Xu, Ling Ye

**Affiliations:** Department of Nephrology, Nanshan Hospital, Shenzhen University, 89 Taoyuan Road, Shenzhen, 518052 P. R. China; Health Science Center, Shenzhen University, Shenzhen, China

**Keywords:** Kimura’s disease, Nephrotic syndrome, Eosinophilia, Immunoglobulin E, Glucocorticoid

## Abstract

**Background:**

Kimura's disease (KD) is a slowly progressing rare, benign inflammatory disorder of the soft tissues. It typically presents as subcutaneous tumor-like nodules, located most frequently in the head and neck region. KD is often accompanied by increased peripheral eosinophilia and elevated levels of serum immunoglobulin (Ig) E. There is renal involvement in approximately 12-16% of KD cases. We report the case of a 23-year-old Chinese man who was found to have KD associated with nephrotic syndrome.

**Case presentation:**

A 23-year-old Chinese man presented with edema in both legs and a mass in ulnar side of his right upper arm on August 8^th^ 2013. Before admission to our hospital, an ultrasound examination revealed swollen lymph nodes in the medial aspect of his right upper arm, proximal to the elbow. The patient was admitted on August 19^th^ 2013 as a result of edema, severe proteinuria, and low serum albumin levels. He had a white blood cell count of 7.7 × 10^9^ cells/L, 48.5% eosinophils, 4+ albuminuria, 24-hour urinary protein excretion 9.3 g, serum protein 50.3 g/L; serum albumin 16 g/L and IgE 1,510 IU/ml. A biopsy of the epitrochlear nodes revealed eosinophilic hyperplastic lymphogranulomatous tissue. A renal biopsy indicated focal segmental glomerulosclerosis (FSGS) (cellular variant) with no infiltration of eosinophil in renal interstitium. The results of immune-staining on the renal biopsy were negative for IgG, IgA, IgM, C3 and C1q. The electron microscopic analysis showed podocyte effacement. His final diagnosis was Kimura's disease associated with nephrotic syndrome. He received methylprednisolone therapy as well as symptomatic treatment, and was discharged with key indicators in normal range on September 17^th^ 2013. During the year following, he had methylprednisolone at a maintenance dose of 8 mg/day, and no relapses occurred up to now.

**Conclusion:**

Methylprednisolone therapy is effective in KD associated with nephrotic syndrome, and long-term administration of methylprednisolone at maintenance dose may be a way to prevent relapses of KD.

## Background

Kimura’s disease (KD) is a rare, chronic inflammatory disorder that affects the lymph nodes, soft tissues, and salivary glands. It occurs predominantly in Asian males with a male to female ratio of approximately 9.1:3.5 [1]. There are about 300 reported cases globally, with most occurring in Asia [2]. The etiology of KD has yet to be resolved.

The characteristic manifestations of KD include subcutaneous tumor-like masses in the head and neck region, as well as subcutaneous nodules with eosinophilic lymphoid granulomas. Multiple organs can be affected, with about 12-16% of patients suffering renal involvement [3]. Indeed, when there is renal involvement, the disease may present initially as a nephrotic syndrome. KD is diagnosed after the exclusion of malignancies (e.g., T-cell lymphoma and Hodgkin’s disease [1,3]) based on the presence of a painless subcutaneous tumor-like mass, increased immunoglobulin (Ig)E levels, eosinophilia and a biopsy that shows the abnormal proliferation of lymphoid follicles and vascular endothelium.

Here, we present the case of a patient with KD associated with nephrotic syndrome characterized by focal segmental glomerulosclerosis (FSGS). We also provide an analysis of the diagnostic strategy for recognizing KD associated with nephrotic syndrome, as well as treatments and methods to prevent the relapse of this condition.

## Case presentation

A 23-year-old Chinese man presented with edema in both legs and a mass in ulnar side of his right upper arm on August 8^th^ 2013. The patient reported that the edema had been present for 11 days and that the mass had been palpable for 3 years, and was first detected in October 2010. According to the patient’s recollection, it had apparently increased in size since June 2013 with accompanying warmth of the overlying skin. The patient had not received any prior treatment for these conditions.

Before admission to our hospital, the patient developed worsened lower limb edema, abdominal distention, and oliguria. Two days later, an ultrasound examination revealed swollen lymph nodes in the medial aspect of his right upper arm, proximal to the elbow. Because the appearance of the swollen nodes was consistent with inflammation, the patient was prescribed cefprozil, which resulted in a reduction in the swelling.

The patient was admitted on August 19^th^ 2013 as a result of edema, severe proteinuria, and low serum albumin levels. Tender epitrochlear nodes that were poorly mobile were palpable in his right upper arm, with the largest being approximately 30 × 20 mm in size in physical examination. Hematology revealed a white blood cell count of 7.7 × 10^9^ cells/L, 48.5% eosinophils, and a platelet count of 172 × 10^9^ platelets/L. Urinalysis revealed 4+ albuminuria and 2+ red blood cells per high power field. His 24-hour urinary protein excretion was 9,3g. A blood panel yielded the following results: total serum protein, 50.3 g/L; serum albumin, 16 g/L; serum cholesterol; 13.36 mmol/L; serum triglycerides, 1.85 mmol/L; high-density lipoprotein, 2.32 mmol/L; and low-density lipoprotein level, 9.37 mmol/L. In immunological tests, IgE level was as high as 1,510 IU/ml (reference value 0-100 IU/ml), with normal C3, C4, IgG, IgA, and IgM (1.54g/L, 0.37g/L, 5.82g/L, 3.69g/L and 1.44g/L respectively).

**Figure 1:**
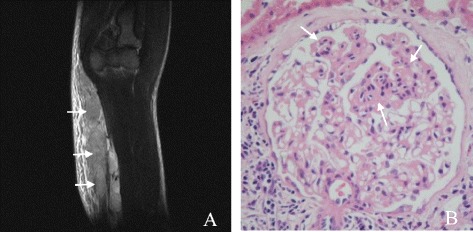
**Lymph node and renal findings. A**: MRI of right arm: a soft tissue density with a vague boundary (arrows) within the medial aspect of the right upper arm, along the brachial artery; **B**: Part of the glomerulus is more heavily stained (arrows) than the other. NO infiltration of eosinophil is found in renal interstitium.

On magnetic resonance imaging (MRI) examination, a soft tissue-density mass was observed within the right medial upper arm, distributed along the brachial artery (Figure 1A). A biopsy of the epitrochlear nodes revealed eosinophilic hyperplastic lymphogranulomatous tissue. A renal biopsy indicated focal segmental glomerulosclerosis (FSGS) (cellular variant) with no infiltration of eosinophil in renal interstitium (Figure 1B). The results of immune-staining on the renal biopsy were negative for immunoglobulin G (IgG), IgA, IgM, C3 and C1q. The electron microscopic analysis showed podocyte effacement. Analysis of a bone marrow sample obtained by aspiration indicated an abnormally high proportion of eosinophils (total eosinophils 14%, metamyelocyte eosinophils, 2%; rod nuclear eosinophils, 2%; and leaf nuclear eosinophils, 10%). A bone marrow biopsy showed a predominance of eosinophils among the granulocytes. The patient had a normal karyotype, and mutation screening from a peripheral blood sample revealed no gene fusions of *FIP1L1-PDGFRα* and *ETV6-PDGFRβ*. No abnormalities were identified at 8p12/8p11.1-q11.1 using fluorescence *in situ* hybridization and the V617F single nucleotide polymorphism of *JAK2*.

The final diagnosis for this patient was KD associated with nephrotic syndrome (FSGS, cellular variant). He was given intravenous methylprednisolone (80 mg/day) for 3 days beginning on August 29^th^, 2013, followed by oral methylprednisolone (36 mg/d, once a day). Meanwhile, he received famotidine (20 mg, twice a day), rocaltrol (0.25 μg, once a day), and caltrate D (300 mg, twice a day) for symptomatic treatment. Six days after the initiation of glucocorticoid treatment, the edema had largely resolved, his serum albumin levels had improved, and his urine was negative for proteinuria. Additionally, the tumor-like mass reduced in size, and his eosinophil ratio and eosinophil counts normalized. Two weeks later, the serum albumin levels had increased to within the normal range and only one soft, smooth, mobile lymph node (5 × 5 mm cross-sectional area) was palpable in his right arm. Therefore, the patient was discharged on September 17^th^ 2013. After a 2-month period of methylprednisolone at a dose of 36 mg/day, the prescribed methylprednisolone dose was tapered by 4 mg every 2 weeks until a maintenance dose of 8 mg/day was reached. During the year following discharge, serum albumin levels and urinalysis test results remained normal and no relapses occurred.

## Discussion

In this report, we described the case of a 23-year-old Chinese man who developed KD with nephrotic syndrome 3 years after the appearance of a tumor-like subcutaneous mass. The subcutaneous mass was located in the right arm, which is atypical for KD.

There is no consensus regarding the optimal treatment for KD. Treatment options include surgical excision, corticosteroids, chemotherapeutic agents, and radiotherapy [4]. Although KD is benign, it is prone to recurrence [5] and can be disfiguring. Corticosteroids are particularly applicable for cases of KD with renal involvement. In the present case, we observed a good response to 3 days of intravenous methylprednisolone (80 mg/day) with subsequent oral methylprednisolone (36 mg/day), and therefore proceeded to taper the methylprednisolone dosage to a long-term maintenance dose (8 mg/day). At the time of writing this report, this regimen appears to have prevented a relapse of KD.

The pathological mechanism of renal involvement in KD is not yet known. Various renal pathologies may present in KD associated with nephrotic syndrome, including minimal change disease [6], mesangio-proliferative glomerulonephritis [7], membranous nephropathy [8], FSGS, IgA nephropathy, IgM nephropathy, and IgE nephropathy. Inexplicably, we observed the complete remission of the nephrotic syndrome in this patient within 6 days of commencing corticosteroid treatment. This is remarkably rapid given that the average remission time for FSGS in primary nephrotic syndrome patients treated with corticosteroids is 4 months.

Rare diseases are likely to be misdiagnosed as doctors lack the required clinical experience. This can often result in a long interval between the onset of symptoms and the correct diagnosis [9], which prevents the initiation of prompt treatment. In this patient, 3 years elapsed before he was diagnosed correctly. To reduce diagnostic errors, enhancing the awareness of rare diseases is necessary for doctors, in addition to having wide-ranging relevant knowledge and critical thinking abilities.

## Conclusions

We describe a successful treatment of Kimura's disease associated with nephrotic syndrome in a patient with methylprednisolone, and prevention of Kimura's disease relapses with long-term administration of methylprednisolone at maintenance dose. Although we cannot conclude long-term administration of methylprednisolone is useful as an effective strategy to prevent relapses yet, our report suggests that further studies are required.

## Consent

Written informed consent was obtained from the patient for publication of this case report and any accompanying images. A copy of the written consent is available for review by the Editor of this journal.

## Abbreviations

FSGS: Focal segmental glomerulosclerosis
Ig: Immunoglobulin
KD: Kimura's disease
MRI: Magnetic resonance imaging
